# Justice and Global Health Research

**DOI:** 10.1080/15265161.2016.1214333

**Published:** 2016-09-21

**Authors:** Sridhar Venkatapuram

**Affiliations:** ^a^ University of Johannesburg and King's College London

One of the most interesting and knotty puzzles in global health justice can be found in Margaret Whitehead's decision tree for determining which health inequalities across human beings demand a social response. Whitehead argued that the subset of health inequalities that are avoidable, unnecessary, and unfair or unjust are health “inequities” (Whitehead 1990). While inequalities are value-free observations, inequities are moral bads that demand social action. While the effort is praiseworthy, I have argued elsewhere that many aspects of the health equity principles are problematic (Venkatapuram 2011). In particular, at the first step, a health inequality or impairment that is deemed unavoidable is moved outside of the scope of equity, ethics, and justice. In philosophy, it is often stated that a moral “ought implies can.” So if a disease/impairment is unpreventable or untreatable, there cannot be an ethical duty to prevent or mitigate a resulting health inequality. However, such a first cut would also dissipate any claims to or duties to conduct research on a disease/impairment that is currently unavoidable because we lack the knowledge about how to do so. A health inequality can also be unavoidable because there are not enough resources in a specific location to apply extant knowledge such as in many developing countries. Therefore, in relation to health and more, the prevailing view has been that moral claims and duties that cannot be immediately fulfilled are meaningless. Informed by his analyses of famines and Ronald Dworkin's account of rights, Amartya Sen offered a novel argument for a “meta-right”—a meta-right to *x* is a right to policies *p*(*x*) that genuinely aim to have the right to *x* realizable (Sen 1984). Sen's meta-right challenges Whitehead's first principle. When a health inequality is deemed unavoidable, a meta-right to health can still motivate both health sciences research and broader social and economic policies that aim toward realizing a right to health. That is why it is at the core of my argument for a human right to health or, more accurately, a meta-right to the meta-capability to be healthy.

It is thus noteworthy that Pratt and Hyder present a justice-based argument and an “ethical checklist” for the governance of transnational global health research consortia (GHRCs) (Pratt and Hyder 2016) They present criteria for what global health research should be done as well as who should do what, where, when, how, and so forth. Importantly, these criteria are said to reflect the capabilities approach (CA) to (global) health justice, particularly relying on Jennifer Prah Ruger's prolific writings on health (care) governance. I am wholly sympathetic with the mission of linking global health research to conceptions of (global) justice, as well as to extending and utilizing the CA for such a mission. However, Pratt and Hyder's argument is unsatisfactory. I focus on three aspects, including their principle of LMICs first and foremost; their priority of worst off in terms of shortfall equity; and their purpose of science.

Throughout the article and in the checklist, Pratt and Hyder assert the importance of the GHRCs ensuring that their LMIC research partners are the center of the research endeavor; their voices and those of their community members must be heard; their priorities should determine the GHRC's priorities; most of the GHRC's resources should go to the LMIC researchers and research; and leadership roles should be given to LMIC researchers. Pratt and Hyder do not provide any justification for why research resources should be directed to LMICs in the first place, or for why GHRCs should be initially formed. They point to the CA for such justification, and focus on arguing for putting LMIC research(ers) first in various aspects of the functioning of the consortia. This is understandable as so much of health equity, human rights, and human-centered development thinking argues for participation of those affected (i.e., “nothing about us without us”). However, an important consideration moves against a uniform central role for LMIC researchers, especially codified into a checklist.

In global justice philosophy, there is a general acceptance of an ethical duty to correct harms one has caused even if it occurs in other countries (Føllesdal and Pogge 2005). It is also recognized that there is an ethical duty to prevent possible future harms from one's actions. Correcting past harms and preventing future harms could address significant human suffering/health inequalities in the world. This could produce duties to conduct global health research and form GHRCs. For actors who appear to have no past or future harm links to people that are suffering in the world, the possible duty to act is based on capacity. (Sen 2009; Singer 2004) One has a moral obligation to consider helping alleviate suffering of individuals wherever one finds them, in light of and commensurate to one's capacity to do so. In other words, one ought to help if one can and to the extent one can. According to Pratt and Hyder's argument, rich country GHRC members may have exceptional capacity but their focus must be on building self-sufficiency of LMIC research partners. Why should improving skills and capacity of LMIC researchers be a stronger moral duty than the duty of helping alleviate suffering of people commensurate with one's exceptional capacity? There is no central tenet of the CA that would privilege LMIC researchers in all aspects. Moreover, GHRCs usually involve LMIC researchers across a number of countries. While the authors focus on the dyadic relationships between LMIC and HIC researchers, what are the procedures and principles for adjudicating disagreements between researchers from different LMICs within the consortia?

Pratt and Hyder argue that research priorities of GHRCs should reflect the needs of the worst off in LMICs. Following Ruger, they define the worst off in terms of those who have the greatest shortfall from the world's highest/best health achievements (Ruger 2010). While it is not clear whether the basic unit is individuals or groups, let's assume they mean groups (contra a central aspect of the CA). If we take Japan, which has the world's highest life expectancy as the standard, then those LMICs or groups within LMICs with the greatest shortfall from that Japanese threshold should get priority in research. However, shortfall equity does not necessarily mean that the standard from which one measures has to be the highest achievable outcome in the world. A sufficient or average threshold could also be used. Pratt and Hyder go with the highest outcomes in human populations. Given that there is reasonable disagreement on whether one kind of impairment is worse than other, or whether staying alive is always good, there would have to be a lot of work done before we could rank which LMICs or groups according to their shortfalls. One could imagine that a measurement such as the disability-adjusted life year (DALY) would be very helpful. It may be plausible to identify what diseases/impairments create the greatest shortfall (most DALYs lost) across countries or across groups within countries. Such ranking could then act as a research priority-setting exercise for GHRCs. However, whether using DALYs or another metric, much more reasoning must be provided for aiming to achieve maximal outcomes. Prioritizing research according to greatest group shortfall presumably is in order to produce research outputs that will have the greatest reductions in health inequalities. Given that the CA was partially born out of the critique of utilitarianism including its central tenet of maximization, it would be inconsistent to aim for maximization in a CA-based argument without giving a clear justification.

A third and important component of Pratt and Hyder's argument is the implicit assertion that the primary aim of GHRCs should be reducing health disparities within and across countries. Given that GHRCs are research coordinating entities, the assertion is that the aim of the scientists involved should be to reduce health disparities. Furthermore, as the checklist is meant for all GHRCs, all scientists involved in any GHRC must do science that aims to reduce health disparities (to the greatest extent). While we may be able to argue that one primary aim of GHRCs should be to reduce health disparities on various grounds, the move to assert that all the research and scientists involved in GHRCs must have a social mission may be unwise. Reconciling the aims and methods of scientific practice with instrumental and social goals such as reducing health disparities requires much more careful reasoning than provided here. The authors are especially vulnerable for relying on Ruger's expositions on governance in order to establish the moral purpose of GHRCs and their scientists. In particular, the debates about the moral purposes of health sciences have recently flourished to the greatest extent in relation to social determinants of health research, or social epidemiology. At least in the articles the authors cite, Ruger expressly excludes social determinants of health from social policy because “we do not know enough.” Ruger's argument regarding research is that the focus on health capability motivates claims to research on health care, broadly understood to include public health goods and services and equitable finance. As a result, Ruger's governance framework is closed off to important ethical arguments for doing science to reduce social inequalities in health while also doing excellent science. Without being informed by these debates or much philosophy of science, Pratt and Hyder's argument and checklist are vulnerable to the charge that the health sciences are being infused with social values and likely to result in bad science.
